# Supplementary material for: Splice-Junction-Based Mapping of Alternative Isoforms in the Human Proteome
Source: Cell Rep. Author manuscript; Available in PMC 2020 Jan 15. (PMC6961840; doi:10.1016/j.celrep.2019.11.026)
Supplement: 2 [file NIHMS1546469-supplement-2.zip › DF1/PXD010154/Testis/Testis_41_LGALS1_GGSLNLKPGECLR.pdf]

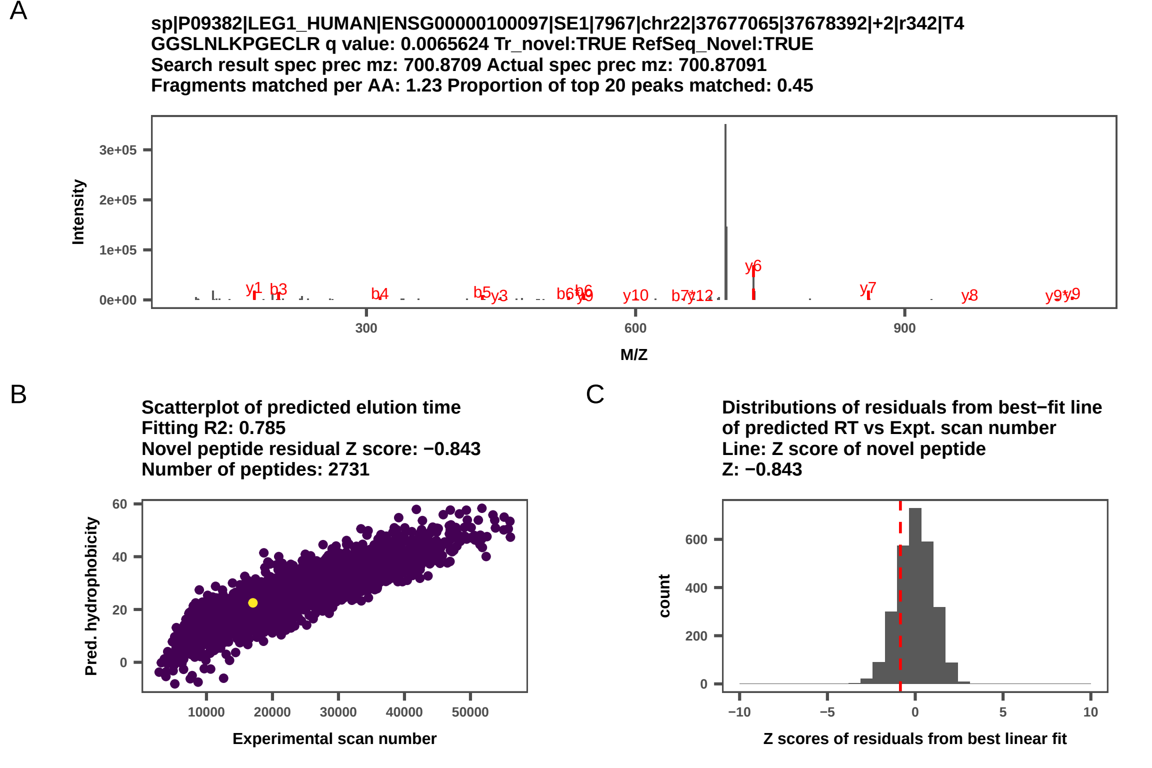
A
sp|P09382|LEG1_HUMAN|ENSG00000100097|SE1|7967|chr22|37677065|37678392|+2|r342|T4
GGSLNLKPGECLR q value: 0.0065624 Tr_novel:TRUE RefSeq_Novel:TRUE
Search result spec prec mz: 700.8709 Actual spec prec mz: 700.87091
Fragments matched per AA: 1.23 Proportion of top 20 peaks matched: 0.45
Intensity
3e+05
2e+05
1e+05
0e+00
y1
b3
b4
b5
y3
y10
y6
y7
y8
300
600
900
M/Z
B
Scatterplot of predicted elution time
Fitting R2: 0.785
Novel peptide residual Z score: −0.843
Number of peptides: 2731
Pred. hydrophobicity
60
40
20
0
10000
20000
30000
40000
50000
Experimental scan number
C
Distributions of residuals from best−fit line
of predicted RT vs Expt. scan number
Line: Z score of novel peptide
Z: −0.843
count
600
400
200
0
−10
−5
0
5
10
Z scores of residuals from best linear fit
